# A Competing Voices Test for Hearing-Impaired Listeners Applied to Spatial Separation and Ideal Time-Frequency Masks

**DOI:** 10.1177/2331216519848288

**Published:** 2019-05-20

**Authors:** Lars Bramsløw, Marianna Vatti, Rikke Rossing, Gaurav Naithani, Niels Henrik Pontoppidan

**Affiliations:** 1 Eriksholm Research Centre, Oticon A/S, Denmark; 2 Laboratory of Signal Processing, Tampere University, Finland

**Keywords:** speech test, hearing impairment, speech masker, spatial hearing, ideal masks

## Abstract

People with hearing impairment find competing voices scenarios to be challenging, both with respect to switching attention from one talker to the other, as well as maintaining attention. With the Danish competing voices test (CVT) presented here, the dual-attention skills can be assessed. The CVT provides sentences spoken by three male and three female talkers, played in sentence pairs. The task of the listener is to repeat the target sentence from the sentence pair based on cueing either before or after playback. One potential way of assisting segregation of two talkers is to take advantage of spatial unmasking by presenting one talker per ear after application of time-frequency masks for separating the mixture. Using the CVT, this study evaluated four spatial conditions in 14 moderate-to-severely hearing-impaired listeners to establish benchmark results for this type of algorithm applied to hearing-impaired listeners. The four spatial conditions were as follows: summed (diotic), separate, the ideal ratio mask, and the ideal binary mask. The results show that the test is sensitive to the change in spatial condition. The temporal position of the cue has a large impact, as cueing the target talker before playback focuses the attention toward the target, whereas cueing after playback requires equal attention to the two talkers, which is more difficult. Furthermore, both applied ideal masks show test scores very close to the ideal separate spatial condition, suggesting that this technique is useful for future separation algorithms using estimated rather than ideal masks.

## Introduction

Competing voices scenarios are especially challenging for a hearing aid user. They might occur, for instance, while attending to two voices in a restaurant or while watching TV and attending to a voice in the room at the same time. For a hearing aid user, a situation as simple as two competing voices next to each other across a table causes much informational masking (Ezzatian, Li, Pichora-Fuller, & Schneider, 2015; Ihlefeld & Shinn-Cunningham, 2008), causing both voices to mask and disturb one another (Brungart, 2001). In these cases, the two voices are both of interest to the user who is struggling to divide attention between both, especially when there is little or no spatial separation between the two. Rather than one voice being the target and one voice being the masker, they both act as masker and target. To test the performance of relevant enhancement algorithms in this user scenario, a new type of speech test has been developed and is presented here.

### Speech Test

Compared with traditional speech tests with a single target voice, a competing voices test (CVT) would have two or more targets that are equally important to follow. In the simplest case, there would be no masker or noise. Concurrent talkers, voice-on-voice, and competing voices scenarios have also been reported in the literature, starting with Cherry (1953). A commonly applied test method is the coordinate response measure (CRM) test in English using simultaneous talkers with a fixed sentence structure, for example, “Ready Charlie go to Blue Five now”: names for cueing and colors and numbers for response options (Bolia, Nelson, Ericson, & Simpson, 2000). The fixed and time-aligned sentence structure of the test is suited for exploring low-level spatial and phonemic cues and has been used for testing spatial hearing and attention (e.g., Best, Gallun, Ihlefeld, & Shinn-Cunningham, 2006; Humes, Lee, & Coughlin, 2006; Ihlefeld & Shinn-Cunningham, 2008), but the structured sentences and the very precise time-alignment do not represent ordinary conversations. More recently, a similar Danish test was presented by Nielsen, Dau, and Neher (2014), using a carrier sentence with two time-aligned open-response words. With three female talkers, a difficult dual-attention task can be implemented, but the low resolution in the word-score count (0, 1, 2) and the lack of context makes the test less relevant for the current purpose. Helfer, Chevalier, and Freyman (2010) also investigated competing voice disturbances, but with a designated target and in a dual-task paradigm using time-reversed maskers. Thus, the competing voices were not equally important. Mackersie, Prida, and Stiles (2001) used pairs of natural sentences and had the listener repeat as much as possible from both sentences, so this was a dual-target, dual-attention task. The segregation skills were then correlated to a simpler psychoacoustic measure of tone fusion in the same hearing-impaired listeners.

In a study quite similar to the present one, Humes et al. (2006) compared selective and divided attention with two competing sentences, using the CRM with different types of cueing and a closed-set response to keywords via a touch screen. Two listener groups were tested in two experiments: elderly hearing-impaired (EHI) listeners and young normal-hearing (YNH) listeners, and generally, they found that the EHI group performed worse than the YNH group. In Experiment 1, two cue types and two spatial modes were used: a call-sign cue for both monaural and dichotic presentation and an ear (side) cue for dichotic presentation. The sentence pairs were created by randomly selecting between three female and three male talkers in either same-gender or different-gender pairs. Selective attention was elicited by cueing the CRM call sign (name) before the sentence playback, and divided attention was elicited by cueing the call sign after the playback. Selective attention yielded higher correct word percentage scores than divided attention in both groups. Regarding cue types, they found the highest scores for ear cue in dichotic presentation and lowest scores using the call-sign cue in monaural same-gender presentation, the latter scores being far below the rest due to the difficulty in segregating the synchronous name call-sign in same-gender sentence pairs. The EHI group scored higher on different-gender than same-gender pairs, while this difference was not present in the YNH group. In Experiment 2, the stimulus uncertainty was varied by keeping one or both talkers more or less constant through a 32-pair block, but always using different-gender pairs and thus talker as a cue. They found little or no effect of talker uncertainty in both selective and divided attention, so a random switch of talker has little effect on the CRM word scores. As discussed earlier, speech tests such as CRM with a highly synchronized structure were considered less relevant for the present study due to the artificial nature of the sentences and the precise word alignment.

More recently, Kelly et al. (2017) presented an Australian-English speech corpus having five male and five female talkers for testing multitalker scenarios. This corpus is based on the U.K. version of the matrix-type speech test (Hagerman, 1982; Kollmeier et al., 2015). The procedure for level adjustment across the entire corpus was described and applied to create a homogeneous speech corpus with a psychometric function as steep as possible for the corpus. Because the matrix test uses a fixed sentence structure, it was also possible to adjust the length of each word and obtain a highly synchronized, but less natural, speech material.

The CVT presented in this article has evolved over a number of iterations and applications using other cue types and different speech material; this was documented in a series of posters (Bramsløw, Vatti, Hietkamp, & Pontoppidan, 2014, 2015, 2016b). Up until the present study, the CVT has used pairs of Danish Hearing in Noise Test (HINT; Nielsen & Dau, 2011) sentences spoken by one male and one female, with a visual Gender (male/female) cue for the listener, presented either before (Pre) or after (Post) the sentence pair playback. It has now been expanded with more talkers and more cue types; further details are given later.

### Spatial Separation

One way of helping the hearing-impaired user in the two-talker competing voices scenario is to unmix (separate) the two voices and add artificial spatial separation, as originally proposed by Cherry (1953). The extreme case of this is to separate the signal mixture and present the two outputs separately to the two ears (e.g., Humes et al., 2006). In this case, there is no need for the user to actively select one of the separated outputs by, for example, a remote control; rather, it should provide better possibilities for attending to one talker or the other voluntarily simply by shifting attention. The CVT should be able to document a benefit by comparing the mixture (sum) of the two talkers to the perfectly separated signals, that is, diotic versus dichotic presentation.

For separation of competing voices and taking inspiration from computational auditory scene analysis (Wang & Brown, 2006), it has been proposed to apply supervised speech separation by using an estimated time-frequency mask (binary or ratio mask; e.g., Han & Wang, 2012; Seltzer, Raj, & Stern, 2000). It has been reported that significant improvement in speech intelligibility can be achieved both for normal-hearing and hearing-impaired listeners with ideal binary masking (Wang, 2008; Wang, Kjems, Pedersen, Boldt, & Lunner, 2009). It has also been claimed that a binary mask provides the better speech intelligibility at the cost of lower sound quality and likewise that a ratio mask provides higher sound quality, as indicated by objective metrics (Wang, Narayanan, & Wang, 2014). See the methods section for a definition of these two mask types.

The aim of the present study was thus twofold: (a) to add more talkers and refine and evaluate the CVT for hearing-impaired listeners and (b) at the same time apply it for a relevant signal processing algorithm. In this case, the effects of dichotic presentation were tested, using ideal separation and two versions of ideal mask separation. The research questions (RQs) and hypotheses were as follows:

RQ1: Does the proposed CVT force the listener to attend to both talkers?

H1: Yes, this can be obtained by cueing the target sentence to the listener after playing the sentence pair.

RQ2: What is the effect of talker gender mixture on performance?

H2: A difference in talker gender (male–female [MF]) is expected to yield higher CVT scores than same gender, due to larger differences in fundamental frequency (e.g., Humes et al., 2006).

RQ3: Can the CVT detect a benefit from ideal separate (dichotic) presentation of the two talkers compared with sum (diotic) presentation, that is, a large spatial contrast?

H3: Yes, as previously shown (e.g., Humes et al., 2006; Ihlefeld & Shinn-Cunningham, 2008).

RQ4: Is there a segregation benefit by applying ideal binary or ratio masks combined with dichotic presentation?

H4: Yes, and the binary mask is expected to provide the highest benefit (e.g., Wang et al., 2014).

Furthermore, the goal was to estimate the reliability of the CVT and its suitability for hearing-impaired listeners. However, the present article is not intended to present the final version of the CVT as only some spatial contrasts were tested here and no other types of speech processing. Future applications of the CVT may lead to other modifications of the test.

The present publication is a prequel to a study in which a speech separation algorithm using deep learning was tested using the presented version of the CVT together with estimated binary and ratio masks (Bramsløw et al., 2018).

## Methods

### Speech Material

The Danish HINT was the chosen speech corpus for the CVT, being an established and well-documented natural sentences speech material (Nielsen & Dau, 2009, 2011). The Danish HINT uses natural everyday sentences each containing five words, spoken by one male talker. The listener is required to repeat as much as possible of the target sentence; this response is open set due to the natural sentences. The entire corpus consists of 13 lists with 20 sentences each: Lists 1 to 10 are suitable for test, while Lists 11 to 13 have higher spread due to sentence complexity, special words, or other reasons (Nielsen & Dau, 2011), so these three lists were proposed for training the listener prior to the actual test.

Because multiple talkers were required, the HINT sentences with the existing male talker (M1) were rerecorded using two new male talkers (M2, M3) and three new female talkers (F1, F2, F3) to provide six talkers in total. As with M1, all talkers were not professional talkers, but ordinary native Danish talkers chosen within the Research Centre staff. The ages ranged from 25 to 53 years. The three male talkers spoke with an average fundamental frequency (F0) of 100, 130, and 155 Hz, and the three females spoke with 200, 172, and 217 Hz. The following equipment was used for recording: Condenser Microphone AKG C 391 B, Microphone Preamplifier IMG STAGELINE MPA-202, Sound Card RME Multiface II, Stationary PC (Windows 7), and sound recording software Audacity.

The recording was conducted as follows: The talker was situated in an audiometry booth with a PC installed with an internally developed software for running the Danish HINT. The microphone was located approximately 60 cm from the talkers’ mouth with a slightly offset axis. The talker would use the speech test software to play the next HINT sentence and then repeat the sentence while trying to use the same intonation and speed as the original talker. This was done to ensure the same vocal quality across all talkers. Each list was recorded in one take and recorded twice to have two versions.

### Sentence Postprocessing

The sentences were evaluated by the first author, and the overall best take of the two, with the most natural speech quality and least artifacts (e.g., coughs, repeats, stuttering), was selected for each sentence. Using the Adobe Audition software, all selected sentences were then manually cut into separate wave files and named according to talker, list, and sentence number. This was done with the constraint that the original male HINT (M1) should not be changed.

An automated way of temporally aligning the manually edited sentences was now applied to minimize temporal talker-specific effects in the test. At the same time, a natural variance of time alignment in the signal files, across sentences within talkers, as in the original HINT, was not changed. Once applied, the alignment was fixed and then represented a compromise that could be used for both the Pre and Post cues. The procedure was as follows: Each of the new sentences was time aligned to the same M1 sentence by calculating the cross correlation between the envelopes of the two signals and then shifting the new sentence accordingly. The negative (backward) time shift was limited to −0.15 s to avoid removal of initial syllable and still maintaining the original M1 files without zero padding. A 200-samples half-Hann window was applied in either ends of the signals to get soft on/off ramps and avoid transients. No time scaling was applied, for example, stretching, so there could still be differences across talkers due to different speaking rates; however, these were not measured.

In the original Danish HINT test, all sentences were level adjusted to make them equally intelligible in stationary, speech weighted noise (Nielsen & Dau, 2009), by using an adaptive procedure based on the listener’s judgment of “ok” intelligibility and following scaling up or down per sentence. As a result, Nielsen and Dau (2009) estimated and applied sentence gains of ± 3 dB for the test lists, resulting in a variation in signal-to-noise ratio across sentences when played in a stationary noise background. In the present study, this procedure should ideally be done for every added talker, but it was decided to use the original M1 HINT as reference and thus not modify sentence levels for that talker. Hence, the original M1 sentence gains were obtained from the authors and applied to the new five talkers, such that a given sentence had the same RMS level as for M1, regardless of talker. This seems appropriate for correcting syntactic and semantic differences but does not compensate for any interactions between talker and sentence. The simpler alternative of using the same RMS for all sentences could also have been applied; however, the approach chosen here does provide some ecologically relevant variations in the signal-to-signal ratio, reflecting real-life variations, while keeping the average at 0 dB. The five new talkers were not spectrally matched to M1, thus making them naturally diverse.

All speech stimuli were presented though Sennheiser HDA200 headphones. In the preceding signal processing, the average equivalent free-field speech level was set at 65 dB sound pressure level (linear frequency weighting) followed by individual per ear linear hearing loss compensation as prescribed by the National Acoustic Laboratories Revised Profound (NAL-RP) gain rule (Dillon, 2012) and free-field compensation for the headphone frequency response. All linear frequency shaping was combined and applied to the signals by using a 256-tap finite impulse response (FIR) filter in the MATLAB application used for administering the test. Thus, no hearing aids were used during the test.

### Test Procedure

Each CVT trial presented sentences in pairs by selecting from two different lists and randomizing the sentence order within lists, that is, 20 sentence pairs per trial. The six talkers were randomly combined in all possible pairs, but in such a way that each trial contained 25% male–male (MM) pairs, 25% female–female (FF) pairs, and 50% male-female (MF) pairs, that is, same amount of same-gender and different-gender pairs. Within each trial, all the included talker pairs were presented in random order, equivalent to the “maximum uncertainty” used by Humes et al. (2006, p2933). In the three dichotic cases, the target talker was furthermore randomized between the left and right ears to make the test as unpredictable as possible for the listener. While this swapping of places and talkers is more complicated and less natural to the listener than a real-life situation, it was designed so to make the predictability as low as possible. The task of the listener was to repeat the target sentence as cued before or after playback (see details later). The listeners were instructed to repeat as many words as possible and were encouraged to guess.

**Figure 1.**
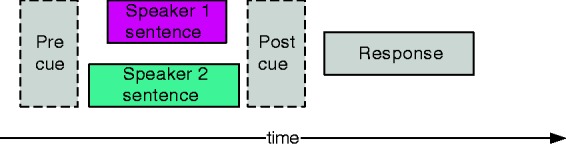
Timeline of one competing voices test item with two sentences played simultaneously in a pair and the cue position either before (Pre) or after (Post) playback.

The cue position was either before playback (Pre) or after playback (Post), equivalent to “selected attention” and “divided attention” (Humes et al., 2006, p2930). The Pre cue condition is similar to a normal target-masker scenario with a preidentified target, whereas the Post cue condition required equal attention to both talkers. This is illustrated in Figure 1.

For both Pre and Post cues, three cue types were tested: Audio, Text, and Gender. The Audio cue was the word “Tomat” (Danish for “tomato”) spoken by the target talker alone in both ears (diotic); thus, the listener had to recognize that voice in the mixture and repeat that target sentence. The Text cue was showing the first or last word from the target sentence on a screen in front of the listener: With Pre cue, it was the first word and with Post cue, the last word. For this cue, the words-correct score was thus in the range 0 to 4. Finally, the Gender cue was used only with the MF mixtures, and the screen was indicating “male” or “female” to identify the target talker.

The words-correct score was chosen as the CVT outcome measure, unlike the sentence-correct score used in the HINT (Nilsson, Soli, & Sullivan, 1994), for two reasons: It provides a higher resolution per sentence (0–5) than the binary sentence score (correct–incorrect), and it will allow analysis of word glimpsing (Best, Mason, Kidd, Iyer, & Brungart, 2015).

### Test Panel

**Figure 2.**
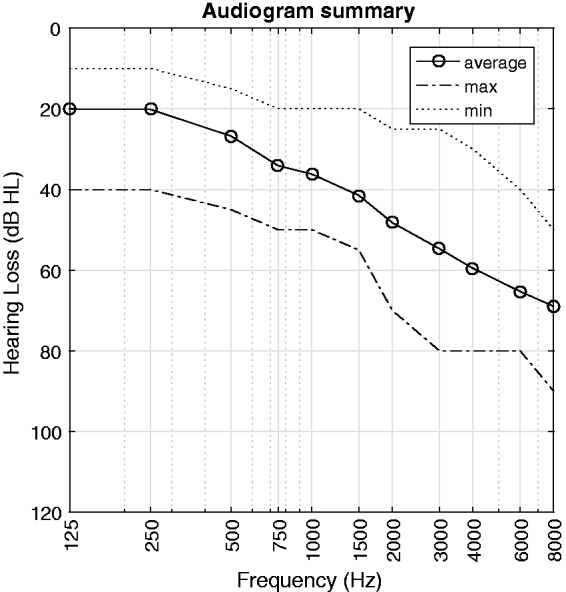
Summary of audiograms for the 14 hearing-impaired test persons. The values are air conduction thresholds across left and right ears.

A total of 14 hearing-impaired persons with moderate, sloping sensorineural hearing loss participated in the test; these specific persons are labeled test persons (TPs) as the experimental (random) factor in the following test design and analysis. The group had 7 males and 7 females, and the age ranged from 68 to 81 years with an average of 73 years. A summary of their air conduction thresholds is shown in Figure 2. The maximum asymmetry across TP, averaged in the 500 to 4000 Hz range, was 12 dB.

As mentioned earlier, individual gain was provided according to the NAL-RP linear fitting rationale. Furthermore, the level of the presentation could be adjusted during the training if requested by the TP: Of 14 TPs, 7 had the level reduced by 4 dB, 2 had it reduced by 2 dB, and 1 had it increased by 2 dB.

All TPs spoke Danish as their first language. The study was approved by the Research Ethics Committees of the Capital Region of Denmark (Reference H-1-2011-033). Prior to the experiment, the TPs had signed an informed consent form, and during the experiment, they were free to withdraw from the experiment at any time. The TPs were not paid for their participation, but they were reimbursed for their travel expenses.

### Spatial Contrasts and Ideal Masks

In addition to the refinement of the CVT, the study had two other RQs: to investigate the effect of the simple spatial contrast sum versus separate presentation (RQ3) and to test the benefit of ideal masks for mixture separation when combined with dichotic presentation of the two outputs (RQ4). Thus, the following four spatial conditions were tested: sum, separate, ideal binary mask (IBM), and ideal ratio mask (IRM).

The two types of masks for the separation were calculated as follows: Each sentence in the talker pair, sampled at 44100 Hz, was converted to a spectrogram using a 440-pt. short-time Fourier transform (STFT) with a Hanning window and 50% overlap, corresponding to 5 ms windows. The ideal masks were calculated by comparing the energy of the two spectrograms in the resulting 100 Hz by 5 ms tiles:

For an acoustic mixture y(t) consisting of sources $s_{1}(t)$ and $s_{2}(t)$, the IBM (Wang & Brown, 2006) corresponding to source $s_{1}(t)$ can be defined as

(1) $$M_{1}(t,f)=\begin{array}{c} \{\begin{array}{cc} 1 & \mathrm{if}|S_{1}(t,f)|\geq |S_{2}(t,f)|\\ 0 & \mathrm{otherwise} \end{array} \end{array}$$

Similarly, the IRM (Huang, Kim, Hasegawa-Johnson, & Smaragdis, 2015) corresponding to source $s_{1}(t)$ is,

(2) $$M_{1}(t,f)=\frac{|S_{1}(t,f)|}{|S_{1}(t,f)|+|S_{2}(t,f)|}$$

where, $S_{1}(t,f)$ and $S_{2}(t,f)$ are STFT spectra corresponding to sources $s_{1}(t)$ and $s_{2}(t)$, respectively. Time and frequency indices are denoted by *t* and *f*. For both mask types, the mask corresponding to source$s_{2}(t)$ is

(3) $$M_{2}^{\mathrm{est}}(t,f)=1-M_{1}^{\mathrm{est}}(t,f)$$

such that the sum of the two masks is always 1. The mask calculation and application was very similar to that used by Naithani et al. (2017), however, with different sample rates and STFT length.

The ideal mask conditions were here used together with dichotic presentation to compare them with the perfectly separated signals and validate the mask architecture before applying estimated masks in a follow-up study also using the CVT (Bramsløw et al., 2018).

### Test Design

A summary of the test design listing all experimental factors and levels is shown in Table 1.

**Table 1.** Summary of Test Conditions in the Factorial Design for the Listening Test.

Experimental factor	Number of levels	Labels
Spatial	4	Sum Separate Ideal ratio mask Ideal binary mask
Cuetype	3	Audio Text Gender
Cueposition	2	Pre Post
Gender	3	Male–Female Male–Male Female–Female
Test persons	14	N/A

The first three conditions—Spatial, Cuetype, and Cueposition—were rotated across TP in a balanced Latin square order, with a total of 4 × 3 × 2 = 24 trials per TP. Furthermore, the gender mix and target location were varied randomly within a given 20-pair trial, such that each trial contained 5 MM pairs, 5 FF pairs, and 10 MF pairs; thus, there were the same number of different-gender and same-gender pairs. Because gender mix could only be one value (MF) for the Gender cue, the total number of combinations was 24 + 24 + 8 = 56. The order of lists across trials was randomized such that no lists were repeated in successive trials. Finally, the sentence order within trials was randomized such that all sentences were equally used and that the initial or last words of the two sentences were different in the Text Cuetype.

## Results

**Figure 3.**
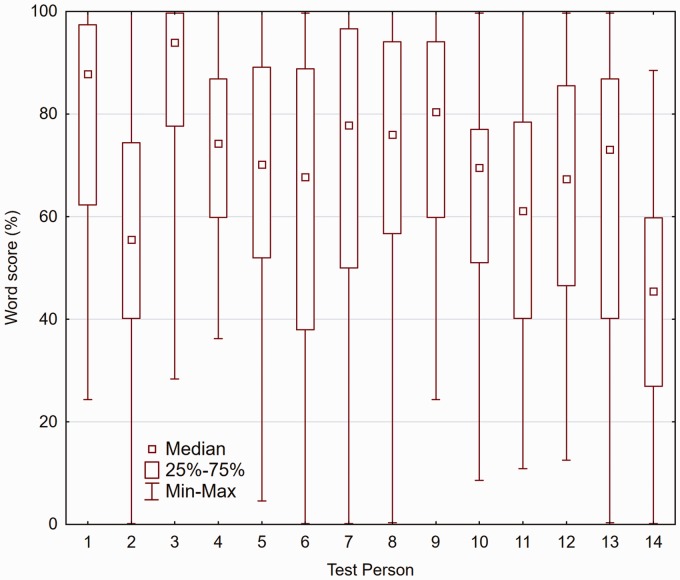
Box-whisker plots for the 14 TP, showing all test conditions.

As described earlier, the outcome measure from each sentence pair was a percent correct word score per sentence pair, based on five words (Audio cue, Gender cue) or four words (Text cue). Within each 20-pair trial, scores belonging to the same experimental combination (see earlier) were averaged. For the purpose of data inspection, these averaged scores—56 data points per TP are shown in Figure 3—a total of 784 data points. The 25% to 75% percentiles are shown as boxes and the min–max values as whiskers. The ranges differ across the 14 TPs. Generally, the full 0% to 100% range is covered, with some TPs operating close to ceiling, while the data from other TPs are more spread out across the full range.

By definition, such 0% to 100% scores are not normally distributed due to floor and ceiling effects as in any psychometric function, and no formal outlier analysis was done. To approximate normal distributions in the following analyses, the data points were therefore rau-transformed (Studebaker, 1985), as is commonly done for speech recognition scores (e.g., Humes et al., 2006).

All rau-transformed data were analyzed using a mixed-model analysis of variance (ANOVA) with TP as a random factor (considered to be random samples from a population) and Gender mix nested under Cuetype (the gender cue can only use the MF combinations). All factors and two-way interactions were included, to check for interactions both between the main experimental factors and the TP factor, that is, to examine whether the different factors were affecting the TPs differently, for instance, whether the Spatial factor was affecting them differently. The ANOVA table is shown in Table 2. All *p* values below .05 are highlighted to indicate statistical significance.

**Table 2.** Summary of Analysis of Variance.

	Effect (fixed/ random)	Nominator *df*	Denominator Syn *df*	*F*	*p*
*Intercept*	*Fixed*	*1*	*12.84*	*494.24*	*.000*
*Spatial*	*Fixed*	*3*	*46.52*	*37.24*	*.000*
*Cuetype*	*Fixed*	*2*	*29.31*	*155.17*	*.000*
*Cueposition*	*Fixed*	*1*	*16.83*	*105.95*	*.000*
*Gender(Cuetype)*	*Fixed*	*2*	*659.00*	*38.21*	*.000*
*Spatial* × *Cuetype*	*Fixed*	*6*	*659.00*	*9.10*	*.000*
Spatial × Cueposition	Fixed	3	659.00	0.73	.532
Cuetype × Cueposition	Fixed	2	659.00	2.73	.066
*Spatial* × *Gender*	*Fixed*	*6*	*659.00*	*4.29*	*.000*
*TP*	*Random*	*13*	*18.10*	*9.28*	*.000*
TP × Spatial	Random	39	659.00	1.28	.122
TP × Cuetype	Random	26	659.00	1.16	.267
*TP* × *Cueposition*	*Random*	*13*	*659.00*	*2.15*	*.010*
Error		659			

*Note*. Significant effects (*p* < .05) are shown in italics. TP = test persons.

All main effects were significant and so were the two-way interactions Spatial × Cuetype and Spatial × Gender (see later for further details). Regarding difference between TPs, the TP main effect and the TP × Cueposition interaction were both significant. It is also interesting to note that there were no significant interactions between Cueposition and the other fixed conditions; the Cueposition was only a main effect, effectively just shifting the word score.

In all plots, the mean values shown are across all other factors than those varied in the plot. Because the design was not balanced, some means were based on different numbers of observations, and thus, the means were calculated as the weighted marginal means (weighted by the respective cell N’s). Likewise, the confidence intervals were 95% confidence intervals of the mean (e.g., Box, Hunter, & Hunter, 1978) and were also calculated across all other factors. Both the weighted means and the confidence intervals were outputs from the general linear model in the STATISTICA v13.3 software. All mean value and confidence interval rau scores were transformed back to percentage scores before plotting, making plots easier to understand. All plots shown in the following have the same axis scale intervals to facilitate a visual comparison of the effect sizes.

**Figure 4.**
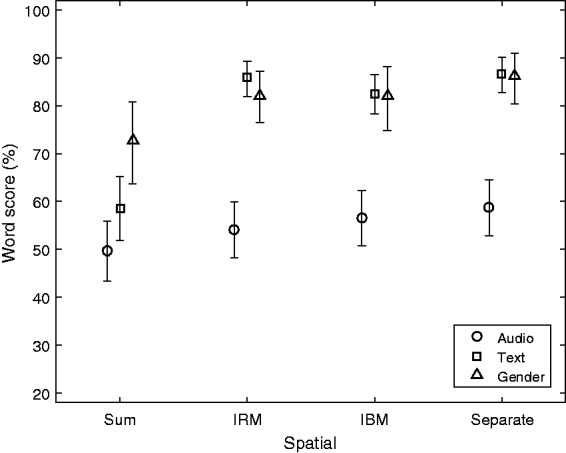
The combined effect of Spatial and Cuetype. Mean values and 95% confidence intervals of the mean are shown. IBM = ideal binary mask; IRM = ideal ratio mask.

The significant main effect of Spatial is not shown by itself, as averaging this across Cuetype has little relevance for the future application of the test. Instead, the combined effects of Spatial and Cuetype are summarized by the interaction effect, shown in Figure 4. The largest sensitivity to the Spatial contrast is observed for the Text Cuetype, with the Sum score at 59% and the three separated conditions around 85%, that is, an effect of approximately 26 percentage points (Tukey honestly significant difference [HSD]: *p* < .001). A smaller, but significant, contrast of 13 percentage points is observed for the Gender cue between Sum and Separate (Tukey HSD: *p* = .033). The Cuetype Audio has no significant differences across the spatial conditions. The overall difference (main effect) among the Cuetypes is also evident from Figure 4 with Audio being at 55%, significantly below the two other types, Text and Gender, on average being around 80% (Tukey HSD: *p* < 0.001). The latter two are not significantly different (Tukey HSD: *p* > .05).

Concerning the effect of Spatial and the Text cue, there is the previously mentioned effect of 26 percentage points between Sum and the three other modes (Tukey HSD: *p* < .001), while the ideal masks (IBM and IRM) are not significantly different from the perfect separation in Separate (Tukey HSD: *p* > .05). The difference between Sum and Separate is 28 percentage points, which is a higher contrast than 23 percentage points obtained in a previous version of the CVT (Figure 3, Bramsløw et al., 2016b).

**Figure 5.**
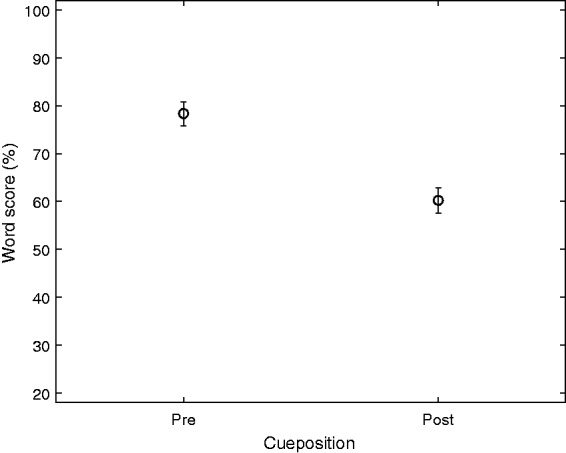
The effect of Cueposition (Pre vs. Post). Mean values and 95% confidence intervals of the mean are shown.

The main effect of Cueposition was also statistically significant, *F*(1, 16.83) = 106.0, *p* < .01, with mean scores at 78% for Pre and 60% for Post as shown in Figure 5. The only significant interaction with Cueposition is the TP interaction (discussed later), indicating that different persons have different benefit by going from Post to Pre.

**Figure 6.**
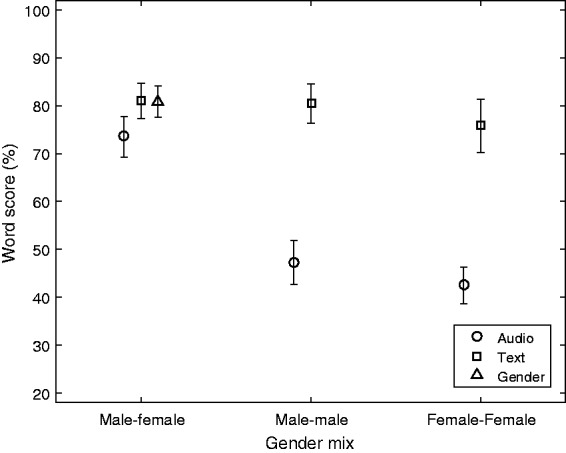
The combined effect of Gender mix and Cuetype. Mean values and 95% confidence intervals of the mean are shown.

Regarding Gender mix and Cuetype, the corresponding results are shown in Figure 6. The Text cue shows no significant effect of Gender mix, while the Audio cue shows a large, significant effect going from 74% down to approximately 45% (Tukey HSD: *p* < .001). The main effect of Gender mix is not shown as it is not relevant to generalize the effect across the three very different Cuetypes.

**Figure 7.**
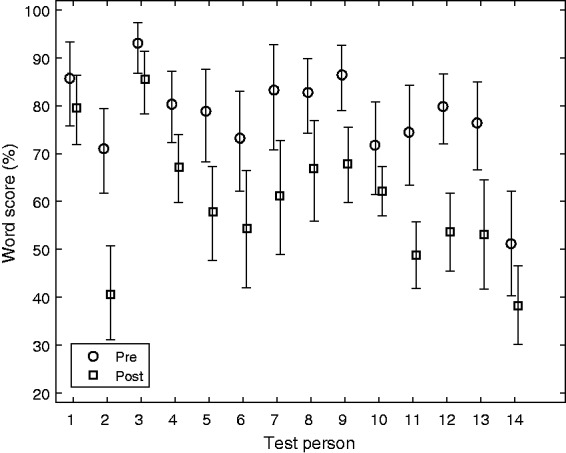
The interaction effect, combining TP and Cueposition (Pre vs. Post). Mean values and 95% confidence intervals of the mean are shown.

In addition to group results, it is also relevant to investigate the spread across the listeners. This is shown in Figure 7 for the significant TP × Cueposition interaction. The interaction can be observed as a large spread in the Pre–Post differences across TPs. The worst performers for the Post cue score around 40%, while the best score for the Pre cue is 93% close to 100%. Averaged across the two Cuetypes, the TPs range from approximately 45% to 90% word score.

## Discussion

The refined CVT was applied to four spatial contrasts. The results are discussed later with reference to the RQs and hypotheses stated in the introduction:

RQ1: Does the proposed CVT force the listener to attend to both talkers?

H1: Yes, this can be obtained by cueing the target sentence to the listener after playing the sentence pair.

The most relevant publication for comparing the present results is Humes et al. (2006). For a careful comparison with the present results, it is important to highlight some important differences in the two studies: The present study used pairs of natural HINT sentences that were not carefully time aligned, together with an open-set response for the entire sentence, whereas Humes et al. (2006) used the time-aligned CRM corpus with closed-set response for number and color. Furthermore, the cue word used here was either the first or last word from the HINT sentences presented before or after sentence pair playback (for Pre and Post cues), whereas Humes et al. (2006) used the first CRM call sign (Name) presented before or after sentence pair playback. This posed an even greater demand on memory and cognitive skills compared with the present study. Finally, the present study has no results for normal-hearing listeners.

To be reliable, the test should clearly indicate to the TP which talker is the target and which is the masker. This seemed to work well for the Text and Gender cues, but the generally poor performance obtained with the Audio cue, as shown in Figure 4, indicates that the two talkers were often confused, which was also remarked by some of the TPs. The confusion appeared to be highest in the two same-gender situations as shown in Figure 6. The text cue, however, is easy to apply in any sentence-based test and is easy and meaningful for the listener to understand, and this cue provided the largest contrast among the Spatial conditions in the experiment as shown in Figure 4.

The present results show higher scores than those obtained with the equivalent “call sign” cues from Humes et al. (2006), but this could be due to the higher context in the HINT sentences than the CRM (which has no context).

Forcing the listener to divide attention across the two talkers was addressed by using the Post cue: From the significant main effect and mean values of 60% and 78% for Pre and Post (Figure 5), respectively, there is clearly a shift in performance. For comparison, the EHI listeners in Humes et al. (2006) had average scores around 80% and 45% for their two dichotic conditions, labeled “selective attention” and “divided attention.”

In the present study, there are no significant interactions with Cueposition, for example, the nonsignificant Cuetype × Cueposition interaction shows no change in Cueposition effect when the Cuetype changes between the three types Audio, Text, and Gender, which could otherwise indicate that the different Cuetypes tap into different degrees of divided attention for the entire group. It might also be that the Cuetype just shifts the difficulty of the test. During discussions in the breaks, some TPs spontaneously indicated that they developed safe strategies, for example, by focusing on one ear, or on the male or female talker (for the Gender cue) or simply attending to the first talker in the pair. The two sentences were not perfectly time aligned—so one talker could lead, and the other could trail by a syllable, depending on the length of the two sentences. By inspecting the TP × Cueposition interaction in Figure 7, large differences among the TPs can be seen, ranging from less than 10 to approximately 30 percentage points, supporting that they used different strategies. Some of the low Post cue scores could indicate an attempt to divide attention rather than to use the safe strategy of choosing one talker, and if this fails, the score is low. Likewise, high Post cue scores indicate a good divided attention because they are significantly above the 50% scores that a safe strategy could provide. With respect to RQ1, the most useful Cuetype was the Text cue, but from the results, it cannot be verified that all TPs were forced to divide their attention between talkers.

Some of the “safe strategies,” focusing on one talker in the Post cue condition, may have been caused by the incomplete temporal overlap between the two sentences. This limitation was difficult to avoid in the present version of the test, when both the Pre and Post cues were used. This could be improved in a future version of the CVT by applying dynamic alignment of either the beginning or end of the two sentences depending on the cue position, and achieve more precise timing, similar to the CRM (Bolia et al., 2000; Humes et al., 2006) or the more recent similar Danish “DAT” test using a carrier sentence with two open-response words (Nielsen et al., 2014).

RQ2: What is the effect of talker gender mixture on performance?

H2: A difference in talker gender (MF) is expected to yield higher CVT scores than same gender, due to larger differences in fundamental frequency.

It could be expected that the MF mixture was easier to segregate due to the difference in fundamental frequency (e.g., Gaudrain, Grimault, & Healy, 2012). The effect of Gender mix can be seen in Figure 6, showing the Gender × Cuetype interaction. For the Text cue, there is no effect of the Gender mix. For the Audio cue, the MM and FF (same-gender) pairs have low scores, indicating that the two voices are easily confused when they are same gender, causing a high risk of missing what the target is.

Thus, for the most sensitive use of the CVT, with the Text cue, there is no effect of Gender mix, and the hypothesis for RQ2 must be rejected in the present experiment. In comparison, Humes et al. (2006) showed a significant advantage of different gender over same gender for the EHI group, strongest in the monaural presentation (similar to the present Sum condition), but also present for dichotic presentation combined with selective attention. For the monaural presentation, the same-gender scores were low for the same reason as in the present study: easy confusion of the two voices.

RQ3: Can the CVT detect a benefit from ideal separate (dichotic) presentation of the two talkers compared with sum (diotic) presentation, that is, a large spatial contrast?

H3: Yes.

Validating the test on the large spatial contrast Separate versus Sum presentation showed that the test is indeed sensitive to this: For the hearing-impaired TPs, there are clear contrasts when using either the Text cue or the Gender cue of roughly 30 percentage points (see Figure 4)—averaged across Pre and Post cue. In comparison, Humes et al. (2006) had a contrast of approximately 25% when comparing dichotic with monaural and likewise averaged across selective attention and divided attention.

RQ4: Is there a segregation benefit by applying ideal binary or ratio masks combined with dichotic presentation?

H4: Yes, and the binary mask is expected to provide the highest benefit.

Two types of ideal masks were applied for separating the mixture and presenting it to the two ears (dichotic presentation): IBM and IRM. The results are shown in Figure 4: Both mask types are not significantly different from the Separate spatial mode. The hypothesis that the binary mask provided higher speech intelligibility than the continuous-valued ratio mask (Wang et al., 2014) was thus not confirmed. In the present study, it was shown that both ideal mask designs do not limit the benefit, and therefore, the same signal processing architecture was used in a subsequent test using nonideal masks estimated by means of deep neural networks for talker separation (Bramsløw et al., 2018).

### Other Findings

It was expected that the CVT should be applicable for EHI listeners without floor and ceiling effects in the outcome measure. The results show that for most conditions, the average score for the entire group is well below the 100% ceiling, according to Figure 4. Because only EHI listeners participated (aged 68–81 years), the effect of age alone cannot be assessed.

As discussed earlier, some listeners may use a “safe strategy,” by which they repeat the first talker or the last talker, in the case of imprecise temporal alignment of the two sentences, or they decide beforehand which ear to attend to. Thus, for Post cue, the average scores may not go much below 50%, and this could be the actual floor for some listeners. However, inspecting the data points and ranges in the box-whisker plots in Figure 3 does not support this suspected floor level, even for the better performers.

When applying a new test method, it is relevant to know the reliability of the measurement, often expressed as the test–retest error. This is often estimated by repeating the entire test and hence have repetition as a separate experimental factor. However, in a large factorial design as the present one, there are many degrees of freedom to estimate this reliability from all higher order interactions which have been pooled into the residual variance in the statistical model, the error mean square (Table 2, bottom line), here equal to 288 rau. Recall that each trial of 20 pairs contained 5 MM pairs, 5 FF pairs, and 10 MF pairs. This means that on average, each gender mix is represented in 20/3 = 6.33 sentence pairs, which also corresponds to one data point in the ANOVA. Based on this, the following estimates of standard deviation can be made:

(4) $$-\text{Per}\;\text{sentence}\;\text{pair}\vdots \mathrm{stddev}=\sqrt{288*\frac{20}{3}}=43.8\text{rau}$$

(5) $$-\text{Per}\;\text{trial}\vdots \mathrm{stddev}=\sqrt{288*\frac{1}{3}}=9.8\text{rau}$$

Hence, the standard deviation for one 20-pair trial is 9.8 rau. When transformed from rau back to percent, this is equivalent to 9.0 percentage points, determined as one standard deviation below the grand mean of all test data (70%) on the psychometric function. This value indicates a small spread per trial, especially given the fluctuation in talker mix, time alignment of the sentences, level variations, and the difficulty of the listening task itself. For comparison, the Danish HINT (Nielsen & Dau, 2011) had a within-subject standard deviation of 0.92 dB and a psychometric-function slope of 14.7% / dB for the unaided hearing-impaired group, which can be translated to a standard deviation of 13.5%. This was for speech in stationary noise and using the adaptive sentence-correct score as in the standard HINT (Nilsson et al., 1994).

In clinical terms, the test is practical and quick to administer. However, when administering the CVT, reuse of the 10 HINT lists occurs very quickly, as each trial uses 2 lists. Therefore, learning will take place (Bramsløw, Simonsen, El Hichou, Hashem, & Hietkamp, 2016a), and this needs to be addressed by proper balancing of the test conditions across TPs.

When applying a new speech test for a given purpose or a new type of listeners, it is relevant to know what the normative results are, that is, the results for a normal-hearing group. For the present version of the CVT, no normal-hearing results were obtained. However, similar data were obtained in an earlier test with four YNH listeners using slightly different test conditions and only the Gender cue with one male and one female talker (Bramsløw et al., 2015). These results were published in Bramsløw et al. (2018): For the Pre cue, the normal-hearing word scores were 97% and 99% for the Sum and Separate spatial modes, thus very close to ceiling, and the difference was nonsignificant. For the Post cue, the scores were 90% and 98% for the Sum and Separate spatial modes. The latter difference was significant (Tukey HSD: *p* = .0003) but indicates that the performance for younger, normal-hearing listeners is near perfect, almost independent of spatial condition. The effect of age versus hearing loss cannot be separated, as the test has not been administered neither to young hearing-impaired listeners nor to elderly normal-hearing listeners.

## Conclusions

The current version of the CVT has been applied to four spatial conditions: Sum, Separate, and two ideal mask separation algorithms. It was able to detect statistically significant differences between the extremes Sum and Separate and also between Sum and two versions of ideal mask separation, and thus it can be used for testing these types of spatial processing in hearing aids.

For the hearing-impaired TPs in the current study, average scores range between 45% and 90%, which is generally between floor and ceiling and thus in the most sensitive range around the 50% score point. This should be compared with normal-hearing TPs, who score close to 100, that is, close to ceiling, as reported in an earlier study (Bramsløw et al., 2015).

The Cuepositions Pre and Post were included in the test design to allow testing a target-masker scenario (selective attention) versus a competing voices scenario (divided attention), and a significant but relative small effect of 18 percentage points was found. In the case of Post cue, it cannot be concluded that divided attention was always elicited by the test or if some listeners employed safe strategies and attended to one ear or one talker giving a 50% chance to choose the right one. The Cueposition did not show different results (interactions) across the other experimental factors, except for TP, showing a significant spread in Pre/Post cue difference across the TP.

Among the three cue types Audio, Text, and Gender, the Text cue was the most sensitive, providing a 28-percentage point contrast between Sum and Separate, compared with previously 23 percentage points (Bramsløw et al., 2016b). The text cue was insensitive to Gender mix and is thus recommended for future applications for both same-gender and mixed-gender talker pairs.

Regarding the test of ideal masks with the given time-frequency resolution, the two ideal masks, Ratio Mask and Binary Mask provide the same word scores as the separated signals. Thus, the time-frequency masking design applied here and assessed with the CVT does not limit the spatial benefit and can be used for testing of realistic non-ideal-mask based speech separation algorithms.
